# Presbycusis Across the Lifespan: Genetic, Molecular, and Multi-Omics Contributions

**DOI:** 10.3390/audiolres16030081

**Published:** 2026-05-26

**Authors:** Anna Morgan, Paolo Gasparini, Giorgia Girotto

**Affiliations:** 1 Department of Medicine, Surgery and Health Sciences, Institute for Maternal and Child Health—IRCCS “Burlo Garofolo”, 34137 Trieste, Italy; paolo.gasparini@burlo.trieste.it (P.G.); giorgia.girotto@burlo.trieste.it (G.G.); 2 Department of Medicine, Surgery and Health Sciences, University of Trieste, 34149 Trieste, Italy

**Keywords:** presbycusis, age-related hearing loss, genetics, noise exposure, audiology, risk factors

## Abstract

Presbycusis, or age-related hearing loss (ARHL), is a multifactorial disorder characterized by a gradual, bilateral sensorineural decline in hearing sensitivity, predominantly affecting high-frequency sounds. It is one of the most common chronic conditions in the aging population and represents a major public health concern due to its high prevalence and progressive nature. Presbycusis significantly impairs speech perception, especially in noisy environments, leading to communication difficulties, reduced social participation, increased risk of social isolation, and a decline in quality of life. Moreover, growing evidence highlights a strong association between ARHL and cognitive impairment, dementia, depression, and increased frailty in older adults. The etiology of presbycusis is complex and involves the interplay between genetic predisposition and cumulative environmental and lifestyle-related factors. Genetic susceptibility influences cochlear aging, neural degeneration, and vulnerability to external insults. Non-genetic contributors include chronic noise exposure, cardiovascular and metabolic disorders such as diabetes and dyslipidemia, ototoxic medications, smoking, and other lifestyle factors that may accelerate cochlear damage through oxidative stress and microvascular dysfunction. This narrative review aims to provide an updated overview of the genetic and environmental determinants involved in the development and progression of presbycusis. Furthermore, it discusses the clinical implications of these factors for early identification, audiological evaluation, prevention strategies, and personalized management approaches. A better understanding of the multifactorial nature of presbycusis may support the development of targeted interventions to preserve hearing function and improve overall health outcomes in the aging population.

## 1. Introduction

Age-related hearing loss (ARHL), commonly referred to as presbycusis, is the most prevalent sensory impairment in older adults and represents a growing global health burden in aging societies [1]. Epidemiological data indicate that more than one-third of individuals over the age of 65 experience clinically significant hearing impairment, with prevalence rising steeply in later decades of life [2]. Beyond its impact on auditory sensitivity, presbycusis is increasingly recognized as a major contributor to social isolation, depression, frailty, cognitive decline, and reduced quality of life [3,4].

Despite its strong association with chronological age, presbycusis exhibits marked interindividual variability. Individuals of similar age and comparable environmental exposure histories often display profoundly different auditory phenotypes, ranging from near-normal hearing to severe impairment. This heterogeneity cannot be explained by age alone and highlights the contribution of genetic susceptibility, biological aging processes, and their interaction with cumulative environmental stressors. As a result, presbycusis is no longer viewed as an inevitable consequence of aging but rather as a biologically complex and heterogeneous condition [5].

From a pathophysiological standpoint, ARHL reflects progressive dysfunction across multiple cochlear subsystems. Degeneration of inner (IHCs) and outer hair cells (OHCs) contributes to elevated auditory thresholds, while alterations in synapses between IHCs and auditory nerve fibers impair neural encoding. Additional involvement of spiral ganglion neurons and the stria vascularis disrupts neural transmission and ion homeostasis, respectively, giving rise to diverse audiological phenotypes. Importantly, suprathreshold auditory deficits—such as impaired speech perception in noise and reduced temporal resolution—often precede clinically detectable threshold shifts, indicating that molecular and neural alterations arise early in the disease course [6].

Recent advances in human genetics, genomics, and molecular biology have fundamentally reshaped current models of presbycusis. Rather than representing isolated cochlear wear-and-tear, ARHL shares molecular hallmarks with systemic aging and neurodegeneration, including mitochondrial dysfunction, oxidative stress, impaired proteostasis, chronic low-grade inflammation, cellular senescence, and epigenetic drift [7]. The cochlea, characterized by exceptionally high metabolic demand and limited regenerative capacity, is particularly vulnerable to these aging-related mechanisms.

In this context, multi-omics approaches—integrating genomic, epigenomic, transcriptomic, proteomic, and metabolomic data—have emerged as powerful tools for elucidating the biological architecture of auditory aging. These approaches provide a systems-level perspective that moves beyond single-gene associations toward convergent molecular pathways and regulatory networks. This review synthesizes current evidence on the genetic and multi-omics determinants of presbycusis, with the aim of informing emerging precision audiology strategies focused on early detection, risk stratification, and personalized prevention.

## 2. Literature Search Methodology

This narrative review aims to provide a comprehensive and integrated overview of the current knowledge regarding the pathological mechanisms underlying ARHL. Particular attention is devoted to genetic factors, oxidative stress, aging-related processes, multi-omics approaches, clinical interventions, and emerging directions for future research.

A non-systematic literature search was conducted across major scientific databases, including PubMed, Google Scholar, and Web of Science, covering publications available up to May 2026. The search strategy combined controlled vocabulary (MeSH terms) and free-text keywords using Boolean operators to optimize both sensitivity and specificity. The following search queries were employed: (“presbycusis” OR “age-related hearing loss”) AND (“genetics” OR “genome-wide association study” OR “GWAS” OR “genetic*”) AND (“oxidative stress” OR “ROS” OR “mitochondria*”) AND (“aging” OR “ageing”) to identify studies related to ARHL pathogenesis; (“presbycusis” OR “age-related hearing loss”) AND (“oxidative stress” OR “ROS” OR “mitochondria” OR “inflammation” OR “mitochondrial dysfunction” OR “apoptosis” OR “cellular senescence”) for studies specifically focused on oxidative stress and related mechanisms; (“presbycusis” OR “age-related hearing loss”) AND (“multi-omics” OR “transcriptomics” OR “proteomics” OR “metabolomics” OR “epigenomics”) for studies investigating multi-omics approaches; and (“presbycusis” OR “age-related hearing loss”) AND (“therapy” OR “intervention” OR “antioxidants” OR “hearing aids” OR “cochlear implants”) for studies addressing therapeutic interventions.

In addition, the reference lists of key articles, recent meta-analyses, and relevant narrative reviews were manually screened to identify further pertinent studies not captured through the primary search strategy.

Studies were considered eligible if they involved adult populations and addressed at least one of the following domains: identification of genetic determinants associated with ARHL; investigation of mechanistic pathways contributing to its pathogenesis, including oxidative stress, inflammation, and aging-related processes; or application of multi-omics approaches aimed at clarifying disease etiopathogenesis. Only studies published in peer-reviewed journals and written in English were included.

Studies were excluded if they focused on pediatric populations, congenital hearing loss, or non-age-related forms of hearing impairment, such as acute, traumatic, or infectious hearing loss. Additional exclusion criteria included case reports, editorials, letters to the editor, conference abstracts without full data, and studies lacking sufficient methodological detail or clearly defined hearing assessment methods. Duplicate publications and studies based on overlapping datasets were also excluded unless they provided additional relevant information.

The included studies were qualitatively evaluated and narratively synthesized in order to identify consistent findings, emerging themes, and areas of ongoing debate or uncertainty. As this was a narrative review, no formal quality assessment or meta-analysis was performed.

## 3. Genetic Architecture of ARHL

### 3.1. Heritability and Polygenic Architecture

Family aggregation studies and twin analyses consistently demonstrate a substantial heritable component of ARHL. Heritability estimates typically range from approximately 35% to 55%, depending on population characteristics, sex, and the specific audiological phenotype assessed [7,8]. These observations indicate that genetic background plays a major role in modulating individual susceptibility to auditory aging, even when environmental exposures are broadly comparable. However, the genetic architecture of presbycusis remains highly complex and only partially resolved. Most associations identified to date derive from candidate-gene studies or genome-wide association studies (GWAS) with relatively modest effect sizes, and many findings have shown limited reproducibility across cohorts. Differences in ethnicity, environmental exposures, audiometric definitions, and reliance on self-reported hearing measures likely contribute to this variability.

Presbycusis does not follow Mendelian inheritance patterns. Instead, it reflects a polygenic architecture in which numerous common genetic variants exert small but cumulative effects on cochlear structure and function. These variants typically involve genes that modulate long-term cellular resilience rather than genes essential for cochlear development. As a result, genetic susceptibility manifests gradually, interacting with biological aging and cumulative environmental stress [9]. Consequently, no single gene can currently be considered either necessary or sufficient to determine ARHL risk, and most identified loci should be interpreted as probabilistic susceptibility factors rather than causal determinants. Genes associated with presbycusis converge on several key biological pathways. One major pathway involves potassium recycling and ion homeostasis, which are essential for maintaining the endocochlear potential. Some examples are genes such as *KCNQ4*, *KCNQ1*, and *KCNE1* which encode potassium channel components expressed in HC and supporting structures [10], or *SLC9A3R1* that contributes to ionic and pH regulation within the cochlear environment [11]. Subtle impairment of these pathways can progressively reduce cochlear efficiency with aging. These genes are biologically plausible candidates because ion transport is fundamental for cochlear physiology, and rare pathogenic variants in some of these genes are known causes of hereditary deafness [12]. A second pathway centers on synaptic transmission and neural integrity. Genes involved in glutamatergic signaling and synaptic architecture, including *GRM7*, *CTBP2*, *SYNJ2*, and *PCDH20*, influence synaptic maintenance, vesicle trafficking, and cell–cell interactions within the auditory pathway [13,14,15]. Among these, *GRM7* is one of the most historically relevant susceptibility loci reported in ARHL studies and has emerged repeatedly in GWAS analyses. However, its individual effect size remains small, and replication has not always been uniform across populations [9,16,17]. Some inconsistencies likely reflect phenotypic heterogeneity, insufficient statistical power, or differences between objective audiometric phenotypes and self-reported hearing impairment. Similarly, *SYNJ2* and *CTBP2* are considered biologically compelling because of their involvement in synaptic maintenance and cochlear neurotransmission, but current evidence still falls short of establishing them as robust predictive biomarkers. Overall, genetic variation in these pathways is thought to increase vulnerability to cochlear synaptopathy, a mechanism increasingly recognized as central to age-related deficits in speech perception and temporal processing [18].

A third functional category encompasses oxidative stress response and mitochondrial metabolism. Genes such as *SOD2*, *CAT*, *GPX1*, and *UCP2* regulate antioxidant defenses and mitochondrial efficiency, thereby modulating cellular resilience to cumulative metabolic stress [19]. Reduced capacity to counteract oxidative damage may accelerate degeneration of HCs, neurons, and strial tissues in genetically susceptible individuals.

Notably, these pathways overlap extensively with mechanisms implicated in systemic aging and neurodegenerative disorders. The convergence of ionic dysregulation, synaptic vulnerability, and oxidative stress supports the view that presbycusis represents an auditory phenotype of broader biological aging rather than an isolated cochlear disorder.

### 3.2. GWAS

GWAS have substantially advanced the understanding of the complex genetic architecture underlying ARHL, demonstrating that this condition is highly polygenic and influenced by numerous common variants, each exerting modest individual effects. Large-scale GWAS conducted in population-based cohorts, including the UK Biobank and subsequent meta-analyses, have identified dozens of genome-wide significant loci associated with adult hearing difficulty [20,21,22]. Among these, genes such as *NID2*, *CLRN2*, *ARHGEF28*, *TRIOBP*, *BAIAP2L2*, *TUB*, *SYNJ2*, and *SPTBN1* have emerged as biologically plausible candidates due to their expression in cochlear structures and their known roles in cytoskeletal organization, synaptic regulation, and auditory HC maintenance [21,22]. Their candidacy is strengthened by known cochlear expression patterns and mechanistic relevance rather than by large statistical effects.

Particularly compelling are associations involving genes already implicated in monogenic deafness syndromes. Variants in *CDH23*, a gene essential for stereocilia tip-link integrity and previously implicated in monogenic deafness, have also been associated with adult hearing difficulty, supporting a continuum model between rare high-penetrance mutations and common low-effect polymorphisms contributing to ARHL susceptibility [22]. Importantly, rare pathogenic variants in *CDH23* likely exert substantially larger effects than typical GWAS single-nucleotide polymorphisms (SNPs), making them biologically more robust despite their lower population frequency. Similar considerations apply to genes such as *MYO6*, *MYO7A*, *TECTA*, and *EYA4*, where rare-variant burden analyses appear to exert larger effect sizes on hearing impairment susceptibility than many common-variant GWAS signals [22]. In general, rare-variant associations tend to provide stronger mechanistic evidence because they more directly alter protein function and often demonstrate clearer genotype–phenotype relationships.

Recent meta-analytic efforts have expanded the catalog of associated loci, identifying up to 48 independent risk variants and highlighting enrichment in genes expressed in the stria vascularis and cochlear lateral wall [20]. Functional annotation of GWAS signals has implicated additional candidate genes, including *KLHDC7B*, *PIK3R3*, *CRIP3*, *MAST2*, and *SLC22A7*, which are involved in intracellular signaling, metabolic regulation, and ion transport pathways relevant to cochlear homeostasis [20,23]. These findings reinforce the concept that age-related auditory decline arises from perturbations across multiple biological systems, including sensory transduction, synaptic integrity, and cellular stress responses. However, several GWAS loci identified in very large cohorts—such as those just mentioned—should be interpreted cautiously. These loci generally exhibit very small odds ratios and are vulnerable to the limitations inherent in hearing-related GWAS, particularly those relying on self-reported hearing difficulty rather than standardized audiometric phenotyping.

Additional associations have been reported for genes including *FBXO2*, *PALM3*, *TWF1*, and *TXNDC17*, suggesting that both known deafness genes and newly implicated loci participate in the genetic architecture of ARHL [22]. These results support a model in which common and rare variants act jointly to influence susceptibility, with rare variants often exhibiting larger effect sizes.

Earlier GWAS conducted in European isolated populations provided foundational evidence that quantitative hearing thresholds have a measurable heritable component and identified novel candidate loci influencing auditory function [24,25]. Subsequent integrative genomic approaches combining GWAS data with transcriptome-wide association analyses (TWAS) have further refined gene prioritization, identifying genes such as *SLC1A6*, *ASTN2*, and *ARF4-AS1* as potential contributors to normal hearing variability and age-related decline through regulatory mechanisms affecting neuronal and cochlear pathways [26]. However, TWAS-based prioritization remains inferential and depends heavily on reference transcriptomic datasets, which are still limited for human cochlear tissues.

More recently, Mendelian randomization studies leveraging GWAS summary statistics have suggested that genetic liability to presbycusis may correlate with structural changes in cortical and auditory brain regions, indicating that ARHL is embedded within broader neurobiological aging processes [27].

A summary of the candidate genes identified through GWAS and discussed in this narrative review is provided in Table 1.

Effect sizes of common variants are generally small, and many associations are based on heterogeneous phenotypes including self-reported hearing difficulty. Rare variants in known deafness genes typically show stronger effect sizes but lower population frequency.

Overall, current GWAS evidence indicates that presbycusis results from the cumulative effects of numerous genetic variants distributed across pathways involved in hair cell mechanotransduction (e.g., *CDH23*, *MYO7A*), synaptic signaling (e.g., *SYNJ2*, *ARHGEF28*), cytoskeletal organization (e.g., *BAIAP2L2*, *SPTBN1*), glutamate transport (e.g., *SLC1A6*), and ion homeostasis (e.g., *SLC22A7*). The continued integration of GWAS findings with functional genomics, transcriptomic profiling, and multi-ethnic cohort studies will be essential to refine causal gene identification and to support the development of predictive biomarkers and targeted therapeutic strategies for ARHL.

However, the explanatory power of currently identified loci remains limited, and no robust polygenic model yet provides clinically meaningful prediction of ARHL risk. A major limitation of many hearing-related GWAS is phenotyping quality. Large resources such as the UK Biobank frequently rely on self-reported “hearing difficulty,” which may encompass heterogeneous conditions including cochlear dysfunction, noise-induced damage, cognitive decline, central auditory processing deficits, depression, or non-cochlear hearing problems. Such heterogeneity substantially reduces phenotypic specificity and increases the likelihood of false-positive or biologically ambiguous associations [28]. Consequently, future progress will depend not only on larger cohorts, but also on more precise audiometric phenotyping, longitudinal study designs, multi-ethnic replication, and functional validation integrating genomics with transcriptomics and cellular biology.

## 4. Molecular Mechanisms Underlying Cochlear Aging

Aging is a multifaceted biological process characterized by progressive molecular and cellular alterations that impair tissue and organ homeostasis, thereby increasing susceptibility to numerous chronic diseases and conditions, including ARHL.

As a natural and complex biological process, aging represents one of the major risk factors for ARHL. Consequently, several hallmarks of aging are also reflected in cochlear aging and may contribute to increased vulnerability to the development and progression of hearing impairment. However, these mechanisms do not act independently; rather, they interact through complex and dynamic pathways, highlighting the need for a holistic and integrative approach to fully understand the pathogenesis of ARHL.

Among the most relevant aging-related processes implicated in cochlear degeneration and ARHL are oxidative stress, mitochondrial dysfunction, cochlear synaptopathy, and neural degeneration.

### 4.1. Oxidative Stress and Redox Dysregulation

Oxidative stress is widely recognized as a central mechanism driving cochlear aging. The cochlea’s exceptionally high metabolic activity, required to sustain mechanoelectrical transduction and ion gradients, generates substantial amounts of reactive oxygen species (ROS) [29]. In particular, mitochondria-rich structures such as HCs, spiral ganglion neurons, and the stria vascularis are major sources of ROS production within the inner ear [30]. Under physiological conditions, ROS are tightly regulated by antioxidant defense systems; however, aging disrupts this balance, leading to cumulative oxidative damage [31,32].

Genetic variation in antioxidant and mitochondrial pathways modulates individual capacity to neutralize ROS and repair oxidative damage. Several genes involved in cellular redox homeostasis have been implicated in cochlear vulnerability, including *SOD1* and *SOD2*, which encode superoxide dismutases responsible for detoxifying superoxide radicals, and *CAT* and *GPX1*, which participate in hydrogen peroxide clearance [33]. In addition, dysregulation of transcriptional regulators of oxidative stress responses, including *NRF2* and its downstream targets, may further impair the activation of endogenous antioxidant defenses during aging [34].

Excessive ROS contribute to lipid peroxidation, protein oxidation, and DNA damage within cochlear cells, ultimately triggering apoptosis or senescence [35]. These effects are amplified by environmental stressors such as chronic noise exposure, metabolic disease, and cardiovascular dysfunction, which further increase oxidative burden and accelerate mitochondrial decline. Notably, mitochondrial DNA damage and impaired respiratory chain function can create a vicious cycle in which ROS accumulation further compromises cellular energy metabolism.

Oxidative stress thus represents a key point of convergence between genetic susceptibility and environmental exposure, providing a mechanistic framework for gene–environment interactions in presbycusis. Variability in antioxidant capacity may help explain inter-individual differences in susceptibility to ARHL despite similar environmental exposures, highlighting redox regulation as a key target for preventative and therapeutic strategies.

### 4.2. Mitochondrial Dysfunction and Energy Failure

Mitochondrial dysfunction constitutes a hallmark of both systemic aging and ARHL. Age-dependent accumulation of mitochondrial DNA (mtDNA) deletions and point mutations has been documented in human cochlear tissues and correlates with degeneration of the stria vascularis and sensory epithelium [36,37]. These alterations impair oxidative phosphorylation, reduce ATP availability, and promote further ROS generation, creating a self-reinforcing cycle of cellular damage.

Several genes involved in mitochondrial maintenance and bioenergetics may modulate susceptibility to cochlear degeneration. Variants affecting mtDNA replication and stability can influence mitochondrial genome integrity and transcriptional efficiency. Likewise, genes regulating mitochondrial dynamics, including *OPA1*, *MFN1*, and *MFN2*, play critical roles in mitochondrial fusion and cristae organization, processes essential for maintaining energetic competence in metabolically active cochlear cells [38]. Defects in mitochondrial quality control pathways, such as impaired mitophagy mediated by *PINK1* and *PRKN*, may further contribute to the accumulation of dysfunctional mitochondria during aging [39].

The stria vascularis is particularly vulnerable to mitochondrial dysfunction due to its role in maintaining the endocochlear potential. Energy failure within strial marginal cells compromises potassium recycling and ion homeostasis, indirectly affecting HC and neuronal function. In addition, mitochondrial impairment in spiral ganglion neurons may exacerbate synaptic vulnerability and contribute to cochlear synaptopathy, a key feature of early auditory aging [40]. Reduced ATP production can also impair ion transporters and gap junction networks required for cochlear electrochemical gradients.

Genetic variants affecting mitochondrial maintenance and quality control may therefore have system-wide effects on cochlear physiology. The interaction between mtDNA damage, impaired mitochondrial turnover, and oxidative stress suggests a tightly interconnected pathogenic axis in which mitochondrial decline amplifies redox imbalance and cellular degeneration. This mitochondrial–oxidative stress interplay provides a mechanistic explanation for the progressive and cumulative nature of presbycusis and highlights mitochondrial pathways as promising targets for therapeutic intervention.

### 4.3. Cochlear Synaptopathy and Neural Degeneration

Cochlear synaptopathy—the loss or dysfunction of synapses between IHCs and auditory nerve fibers—has emerged as a possible central molecular substrate of presbycusis. Studies conducted in the CBA/CaJ mouse model have shown that synaptic degeneration may occur early in the aging process and in the absence of significant HC loss or permanent threshold elevation [41].

Age-related synaptopathy preferentially affects low-spontaneous-rate auditory nerve fibers, which are critical for encoding sounds in noisy environments. Loss of these fibers results in impaired speech perception in noise and reduced temporal coding, hallmark features of presbycusis that are poorly captured by pure-tone audiometry. Progressive synaptic loss is often followed by secondary degeneration of spiral ganglion neurons, further compromising auditory information processing [42].

Nevertheless, much of the current evidence derives from animal and preclinical studies, whereas longitudinal and mechanistic human data remain limited. Therefore, although cochlear synaptopathy and neural degeneration are increasingly considered important components of cochlear aging, their precise causal role, temporal progression, and specificity in ARHL have yet to be fully established.

### 4.4. Central Auditory and Cognitive Mechanisms in ARHL

Although cochlear degeneration represents a major substrate of presbycusis, increasing evidence suggests that age-related hearing difficulties cannot always be fully explained by peripheral dysfunction alone. Alternative frameworks have highlighted the contribution of central auditory aging and cognitive–auditory interactions to speech perception deficits and listening difficulties in older adults, particularly in acoustically challenging environments [43,44].

Central auditory aging models propose that age-dependent alterations within auditory brainstem and cortical pathways—including impaired temporal processing, reduced inhibitory neurotransmission, and maladaptive cortical reorganization—may contribute substantially to deficits in speech-in-noise perception and auditory scene analysis, even in individuals with relatively preserved audiometric thresholds [45]. In parallel, cognitive–auditory interaction frameworks emphasize the role of higher-order cognitive systems, including attention, executive function, and working memory, in compensating for degraded auditory input [46].

Within this perspective, age-related sensory decline may increase listening effort and cognitive load, leading to compensatory recruitment of frontal and attentional neural networks. This interaction between peripheral auditory dysfunction and cognitive processing may partly explain the marked inter-individual variability observed in communicative performance among older adults with similar audiometric profiles [47].

Importantly, these central and cognitive models should not be considered mutually exclusive with peripheral cochlear mechanisms. Rather, current evidence supports a multidimensional interpretation of ARHL in which peripheral degeneration, neural dysfunction, and cognitive aging interact dynamically across the lifespan. Such an integrative framework may help explain the clinical heterogeneity of presbycusis and identify novel targets for diagnostic and therapeutic strategies.

## 5. Multi-Omics Approaches to Presbycusis

### 5.1. Transcriptomics of the Aging Cochlea

Transcriptomic profiling has provided critical insights into age-dependent changes in cochlear gene expression. Studies in animal models and limited human tissues reveal dysregulation of genes involved in mitochondrial metabolism, synaptic maintenance, inflammatory signaling, and apoptotic pathways [48,49,50]. For example, reduced expression of mitochondrial regulators such as PGC1α (*PPARGC1A*) and oxidative phosphorylation genes has been associated with declining bioenergetic capacity [51], while altered transcription of synaptic genes including *OTOF* and *SLC17A8* (VGLUT3) suggests impaired neurotransmission at the inner hair cell–afferent synapse [52]. In parallel, upregulation of pro-apoptotic and stress-responsive genes such as *CASP3* and HSP family members reflects declining cellular resilience and impaired repair capacity in the aging cochlea [53].

Single-cell RNA sequencing has further refined this understanding by revealing cell-type-specific aging trajectories. HCs, supporting cells, spiral ganglion neurons, and strial cells exhibit distinct transcriptional responses to aging, indicating that genetic susceptibility may act through highly cell-specific molecular programs. For instance, HCs show transcriptional signatures consistent with oxidative stress and cytoskeletal remodeling [52,53,54], whereas supporting cells display changes in extracellular matrix and gap junction genes [53,55]. Strial marginal cells exhibit altered expression of ion transporters such as *KCNQ1* and *ATP1A1*, consistent with declining endocochlear potential [56]. These findings highlight that cochlear aging is not uniform but reflects heterogeneous molecular programs across cellular compartments.

### 5.2. Epigenomics and Biological Aging

Epigenetic regulation plays a crucial role in mediating the long-term effects of environmental exposure and metabolic stress on gene expression. Age-related epigenetic drift, characterized by progressive changes in DNA methylation patterns and chromatin accessibility, has been observed in auditory tissues of mouse models as well as in human peripheral blood [57,58,59]. Differential methylation has been reported in genes involved in oxidative stress responses, inflammation, and neuronal signaling and synaptic regulatory genes, suggesting that epigenetic remodeling may modulate pathways already implicated in cochlear aging.

Importantly, epigenetic age acceleration—estimated using DNA methylation clocks such as the Horvath clock—has been associated with poorer auditory function and increased risk of age-related hearing impairment [60]. These findings support the concept that presbycusis reflects biological rather than chronological aging and position epigenetic markers as potential indicators of cochlear aging trajectories. In addition, environmentally induced epigenetic modifications, including those triggered by noise exposure, smoking, or metabolic dysfunction, may create long-lasting transcriptional reprogramming that influences susceptibility to auditory decline later in life.

### 5.3. Proteomics and Metabolomics

Proteomic approaches in mouse models applied to ARHL have begun to reveal distinct protein expression changes in cochlear tissues, including alterations in pathways relevant to cytoskeletal transport, lipid metabolism, and inflammatory signaling that are consistent with age-linked cellular decline [61]. Metabolomic profiling of cochlear tissues has further implicated dysregulation of energy metabolism, purine and amino acid pathways, and lipid oxidation in aged cochlea with hearing loss, suggesting compromised mitochondrial efficiency and altered redox homeostasis in auditory aging [62]. These proteomic and metabolomic signatures provide molecular evidence for antioxidant dysregulation and metabolic perturbations as contributors to presbycusis and highlight the potential of multi-omic biomarkers to reflect cochlear aging processes.

While substantial progress has been achieved through genomics, transcriptomics, epigenomics, proteomics, and metabolomics, a major challenge in presbycusis research remains the integration of these molecular layers into coherent mechanistic models of cochlear aging. Most current studies investigate omics datasets independently, whereas ARHL likely emerges from dynamic interactions across regulatory, transcriptional, metabolic, and cellular systems. A systems-level perspective is therefore necessary to move beyond descriptive associations toward causal biological interpretation.

In ARHL, common and rare genetic variants identified through GWAS and exome sequencing may influence cochlear aging through effects on gene regulation, cellular stress responses, and tissue-specific vulnerability. Integrative approaches combining GWAS signals with expression quantitative trait loci mapping and TWAS could provide an important strategy for prioritizing candidate genes and identifying causal pathways. Integrating these datasets helps distinguish biologically meaningful loci from statistically significant but functionally uncertain associations.

Actually, the different omics approaches across these studies appear to corroborate one another, suggesting that specific signaling pathways play a role in ARHL. Although some samples were derived from different species, these findings may further indicate that the same signaling pathways contribute similarly to the development of ARHL in both mice and humans.

## 6. Gene–Environment Interactions Across the Lifespan

Presbycusis arises from dynamic and cumulative interactions between genetic susceptibility and environmental exposures occurring across the lifespan. While genetic background establishes an individual’s baseline vulnerability to cochlear aging, environmental and lifestyle factors modulate the timing, severity, and phenotypic expression of auditory decline. These interactions converge on shared molecular pathways, including oxidative stress, mitochondrial dysfunction, inflammation, and impaired cellular repair mechanisms.

Chronic noise exposure represents one of the most extensively studied environmental risk factors for ARHL. Even at levels insufficient to cause immediate threshold shifts, repeated noise exposure accelerates cochlear aging by increasing oxidative burden and exacerbating synaptic vulnerability [63]. Genetic variation in pathways regulating redox balance, synaptic maintenance, and calcium homeostasis modulates individual susceptibility to noise-induced cochlear damage, providing a biological basis for the wide interindividual variability observed in noise-related auditory outcomes [64].

Metabolic and cardiovascular factors further interact with genetic susceptibility to influence presbycusis risk. Diabetes, dyslipidemia, and hypertension contribute to cochlear microvascular dysfunction, chronic inflammation, and mitochondrial stress, thereby amplifying age-related degeneration of sensory and neural elements [65]. These systemic conditions share molecular pathways with cochlear aging, reinforcing the concept that presbycusis reflects both local cochlear pathology and broader systemic aging processes.

Ototoxic medications, including aminoglycoside antibiotics and platinum-based chemotherapeutic agents, represent additional environmental modifiers of genetic risk. Subclinical cochlear damage induced by ototoxic exposure may reduce long-term cochlear resilience, lowering the threshold for age-related decline later in life [66]. Genetic variation affecting drug metabolism, mitochondrial maintenance, and antioxidant capacity likely influences individual vulnerability to these agents.

Collectively, gene–environment interactions provide a mechanistic framework for understanding why presbycusis manifests differently across individuals and populations. This framework underscores the importance of integrating genetic data with detailed exposure histories in both research and clinical contexts.

## 7. Implications for Precision Audiology

The growing body of genetic and multi-omics evidence supporting the biological heterogeneity of presbycusis has stimulated interest in the concept of precision audiology. Traditional audiological models, which rely primarily on pure-tone thresholds and chronological age, provide limited insight into the molecular diversity of auditory aging. In contrast, a precision framework seeks to integrate molecular risk factors with functional measures to enable individualized prediction, prevention, and intervention strategies. However, the clinical application of such approaches remains largely exploratory and is not yet ready for routine implementation.

Polygenic risk scores derived from GWAS data offer a quantitative approach to estimating individual susceptibility to accelerated auditory decline. While current models explain only a modest proportion of variance, they demonstrate the feasibility of stratifying populations according to genetic risk [67]. As genomic datasets expand and incorporate more refined phenotypes, polygenic models are theoretically expected to improve in predictive accuracy and clinical relevance.

Similarly, multi-omics biomarkers further enhance the potential for early detection and risk stratification. Epigenetic markers of biological aging, transcriptomic signatures of cochlear stress, and proteomic or metabolomic indicators of oxidative and inflammatory burden may identify individuals at risk before the onset of clinically significant hearing loss. Nevertheless, several major challenges limit their translational applicability. Cochlear tissue is largely inaccessible in living humans, meaning that most studies rely on peripheral blood or surrogate tissues whose relationship to inner-ear biology remains uncertain. In addition, many proposed biomarkers lack reproducibility across cohorts, have not undergone longitudinal validation, and are influenced by systemic aging, comorbidities, medication use, and environmental exposures. Technical complexity, high costs, and limited standardization further constrain their immediate applicability in routine clinical practice. Importantly, a precision audiology framework does not diminish the role of environmental modification. Instead, it enables targeted counseling by identifying individuals who may derive the greatest benefit from noise avoidance, metabolic risk control, or ototoxicity monitoring. By aligning genetic susceptibility with modifiable risk factors, precision audiology emphasizes prevention as a central component of auditory health in aging populations.

## 8. Conclusions

**Figure 1:**
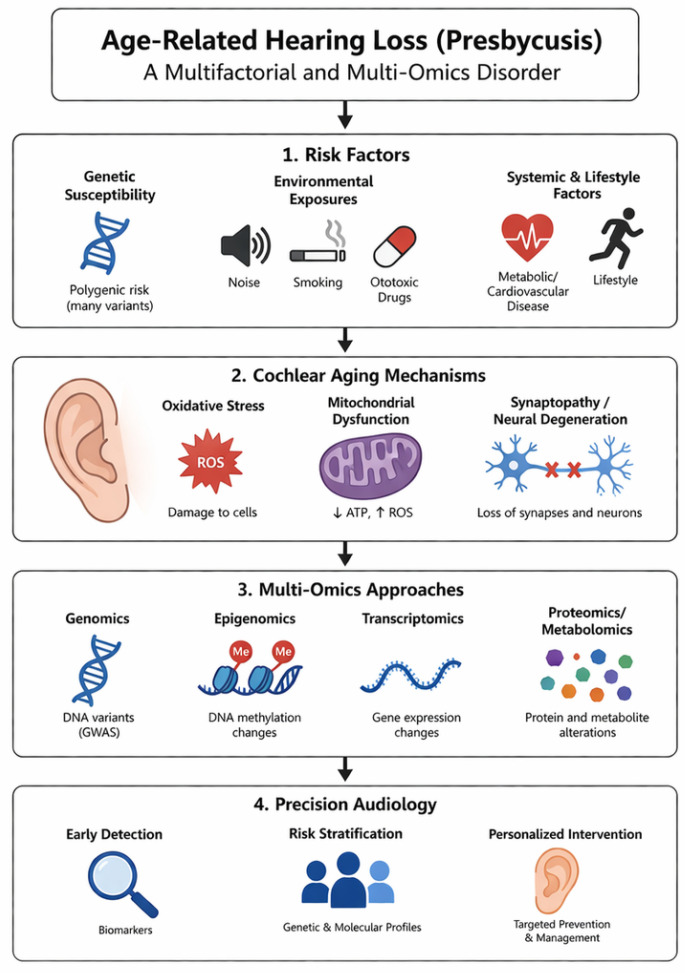
The figure summarizes the mechanisms underlying ARHL and the future potential therapeutic approaches.

Presbycusis is a polygenic and molecularly complex condition arising from the interplay between genetic susceptibility, biological aging processes, and cumulative environmental exposures. Advances in genomics and multi-omics technologies have transformed the understanding of age-related hearing loss, revealing convergent pathways involving oxidative stress, mitochondrial dysfunction, synaptic degeneration, and epigenetic regulation (see Figure 1).

Rather than representing an isolated cochlear disorder, presbycusis shares fundamental mechanisms with systemic aging and neurodegeneration. This convergence highlights the value of systems-level approaches that integrate genetic, molecular, and environmental data to capture the full biological architecture of auditory aging.

The integration of genetic and multi-omics insights into audiological research and clinical practice provides a foundation for precision audiology. By enabling early risk stratification, targeted prevention, and personalized intervention, precision approaches hold promise for preserving auditory function and improving quality of life in aging populations.

## Figures and Tables

**Table 1:** Genetic variants associated with age-related hearing loss (ARHL) and implicated biological pathways.

Biological Pathway	Genes/Loci Identified (GWAS & Related Studies)	Proposed Cochlear Function/Mechanism	Evidence Strength/Notes
Potassium ion homeostasis & endocochlear potential maintenance	*KCNQ4*, *KCNQ1*, *KCNE1*, *SLC9A3R1*	Regulation of potassium recycling, maintenance of endocochlear potential, ionic and pH balance in cochlear fluids	Strong biological plausibility; some genes (e.g., *KCNQ4*) also cause monogenic deafness; GWAS evidence modest and effect sizes small
Synaptic transmission & neural integrity	*GRM7*, *SYNJ2*, *CTBP2*, *PCDH20*, *ARHGEF28*	Synaptic maintenance, glutamatergic signaling, vesicle recycling, cochlear synaptopathy	*GRM7* repeatedly identified in GWAS but with inconsistent replication; overall small effect sizes and phenotypic heterogeneity
HC mechanotransduction & structural integrity	*CDH23*, *MYO7A*, *MYO6*, *TECTA*, *TRIOBP*, *BAIAP2L2*, *SPTBN1*	Stereocilia structure, tip-link integrity, cytoskeletal organization, mechanotransduction	Mix of rare high-impact variants (monogenic deafness genes) and common GWAS signals; rare variants show stronger effect sizes
Oxidative stress response & mitochondrial function	*SOD2*, *CAT*, *GPX1*, *UCP2*	Detoxification of reactive oxygen species, mitochondrial resilience, protection against metabolic stress	Evidence mainly from biological plausibility and expression data; GWAS support indirect
Ion transport & cochlear homeostasis (lateral wall/stria vascularis)	*SLC22A7*, *KLHDC7B*, *PIK3R3*, *CRIP3*, *MAST2*	Regulation of ion transport, metabolic signaling, cochlear fluid homeostasis	Identified in large GWAS/meta-analyses; generally small effect sizes; functional roles still under investigation
Lipid metabolism/intracellular signaling	*FBXO2*, *PALM3*, *TWF1*, *TXNDC17*	Protein turnover, cytoskeletal dynamics, cellular stress response	Emerging associations; limited replication and functional validation
Neurodevelopmental/regulatory pathways (TWAS-inferred)	*SLC1A6*, *ASTN2*, *ARF4-AS1*	Glutamate transport, neuronal signaling, regulatory RNA mechanisms affecting auditory pathways	Inferred from TWAS; dependent on transcriptomic reference datasets; needs functional confirmation

## Data Availability

No new data were generated or analyzed in this study, as this article is a narrative review.

## Abbreviations

The following abbreviations are used in this manuscript:

ARHL	Age-related hearing loss
IHC	Inner Hair Cell
OHC	Outer Hair Cell
SNP	Single-Nucleotide Polymorphism
GWAS	Genome-Wide Association Studies
TWAS	Transcriptome-wide association analyses
ROS	Reactive oxygen species
mtDNA	Mitochondrial DNA
